# An Age-Associated Decline in Thymic Output Differs in Dog Breeds According to Their Longevity

**DOI:** 10.1371/journal.pone.0165968

**Published:** 2016-11-08

**Authors:** Angela Holder, Stephanie Mella, Donald B. Palmer, Richard Aspinall, Brian Catchpole

**Affiliations:** 1 Department of Pathology and Pathogen Biology, Royal Veterinary College, North Mymms, Hertfordshire, United Kingdom; 2 Department of Comparative Biomedical Sciences, Royal Veterinary College, London, United Kingdom; 3 Health and Wellbeing Academy, Postgraduate Medical Institute, Anglia Ruskin University, Chelmsford, Essex, United Kingdom; Colorado State University, UNITED STATES

## Abstract

The age associated decline in immune function is preceded in mammals by a reduction in thymic output. Furthermore, there is increasing evidence of a link between immune competence and lifespan. One approach to determining thymic output is to quantify signal joint T cell receptor excision circles (sj-TRECs), a method which has been developed and used in several mammalian species. Life expectancy and the rate of aging vary in dogs depending upon their breed. In this study, we quantified sj-TRECs in blood samples from dogs of selected breeds to determine whether there was a relationship between longevity and thymic output. In Labrador retrievers, a breed with a median expected lifespan of 11 years, there was an age-associated decline in sj-TREC values, with the greatest decline occurring before 5 years of age, but with sj-TREC still detectable in some geriatric animals, over 13 years of age. In large short-lived breeds (Burnese mountain dogs, Great Danes and Dogue de Bordeaux), the decline in sj-TREC values began earlier in life, compared with small long-lived breeds (Jack Russell terriers and Yorkshire terriers), and the presence of animals with undetectable sj-TRECs occurred at a younger age in the short-lived breeds. The study findings suggest that age-associated changes in canine sj-TRECs are related to breed differences in longevity, and this research highlights the use of dogs as a potential model of immunosenescence.

## Introduction

With increasing age, there is a gradual deterioration in immune function, leading to a reduced response to infectious agents and vaccination, alongside an increase in prevalence of autoimmune and neoplastic diseases [1, 2]. A similar age-related decline in health is seen comparing humans and companion animals [3]. Intimately linked with the decline in immune function is the age-associated regression of the thymus. Since thymic involution is seen in all mammalian species, this has led to the suggestion that immunosenescence, associated with a decline in thymic output, is an evolutionary conserved event [4]. Thus, identifying the features of immunosenescence in companion animals represents an opportunity for comparative and translational research into how immune function declines with age [5, 6].

Thymic involution is associated with a progressive decline in T cell output to the peripheral lymphocyte pool [7] and as a consequence, expansion of existing memory T cell populations can take place [8, 9]. However, this might lead to reduced diversity in the T cell repertoire and impairment of immune responses to novel antigens [9], for example during infection [10] or vaccination [11]. Preservation of immunity is a major contributory factor for maintaining health into old age and although there is evidence for an association between thymic output and longevity, many of these experiments have been performed in inbred or genetically-modified laboratory rodents [12], which might not reflect the situation in humans. Whilst thymic size may be an indicator of immunocompetence/immunosenescence in mammals it is not easily measured clinically. Evaluation of thymic output in terms of the presence of recent thymic emigrants (RTE) in peripheral blood might be an acceptable surrogate marker [13].

During T cell development in the thymus, the T cell receptor δ gene segments, positioned within the TCR α locus, [14, 15] are excised and form a signal joint T cell receptor excision circle (sj-TREC), as a prelude to VDJ recombination. Since its formation occurs specifically in the thymus and this DNA does not replicate, sj-TREC has been used as a marker for RTEs in peripheral blood samples [16, 17]. Although this biomarker has been used to investigate thymic output in several species other studies suggest that the number of T cells containing sj-TRECs might also be influenced by the homeostatic proliferation and apoptosis of naïve T cells in the peripheral lymphocyte pool [18]. In humans [16] and mice [19], real-time qPCR has been employed, showing an age-associated decline in sj-TREC values in blood, suggesting a reduction in the number of RTEs with increasing age. However, sj-TRECs are still detectable, even in some very elderly humans, suggesting that thymic output can be maintained into old age in some individuals [20].

A recent study has demonstrated that sj-TRECs can be measured in companion animals [21], although no significant correlation between sj-TREC values and age was identified in the small group of mixed breed dogs examined, and the relationship between sj-TREC and thymic size was not investigated. Studies in pedigree dogs have demonstrated that there are breed-related differences in longevity [22, 23], the rate of aging [24, 25], and susceptibility to diseases associated with aging [23, 26]. In the UK, the median life expectancy of a Labrador retriever dog is approximately 11 years of age, whereas small breed dogs (such as Jack Russell terriers) are expected to live to around 13 to 14 years of age, compared with large breed dogs (such as Bernese mountain dogs) that die at a median of 6 to 7 years [22, 23]. Such differences in longevity suggest that there are likely to be breed effects/genetic factors influencing the aging process that might impact on the onset of immunosenescence. The aim of the present study was to develop a real-time qPCR assay to measure sj-TRECs in canine blood samples and to examine how age and breed influence sj-TREC values in dogs.

## Materials and Methods

### Study population and samples

Blood samples were identified in the clinical archive of the Royal Veterinary College, University of London, from Labrador retriever dogs (n = 150), Jack Russell terriers (n = 117), Yorkshire terriers (n = 108), Burnese mountain dogs (n = 47), Great Danes (n = 55) and Dogue de Bordeaux (n = 48) that had been referred to the Queen Mother Hospital for Animals. EDTA blood had been archived following completion of diagnostic testing, with ethical approval (approval number URN2016/1475) and informed owner consent for their use in clinical research. Ten to 15 blood samples for each age group were selected, with equal proportions of male and female dogs, where available. All samples were from dogs with a normal lymphocyte count (1.0–4.8 ×10^9^/L), and where possible a normal haematology profile for the other blood cells (5.5–8.5 ×10^12^/L red blood cells, 6.0–17.1 ×10^9^/L white blood cells, 3.0–11.5 ×10^9^/L neutrophils, 0.15–1.5 ×10^9^/L monocytes), as determined by an automated haematology analyser (Siemens ADVIA 2120i).

Anonymised residual EDTA blood samples from Labrador retrievers (n = 30) were provided by IDEXX Laboratories (Wetherby, UK) after completion of diagnostic testing. IDEXX Laboratories has approval for utilising residual clinical samples for development of diagnostic assays, provided that UK data protection legislation is observed. These specific samples had previously been submitted from Companion Care first opinion practices and had been tested as part of a Senior Wellness Programme. The Companion Care Venture Capital Partnership gave consent for the samples to be used for clinical research and project-specific approval was granted from the RVC Ethics and Welfare Committee (approval number: URN2013/1195). All the samples used came from dogs with a normal lymphocyte count (1.4–4.9 ×10^9^/L), and where possible a normal haematology profile for the other blood cells as determined by an automated haematology analyser (XT2000i, Sysmex UK Ltd, Milton Keynes, UK).

### Primer design

Gene specific primers and labelled probes were designed by adapting those used in a human sj-TREC assay [27], which were modified to be specific for the canine orthologue, based on sequence information available in the CanFam3.1 dog genome assembly (http://www.ncbi.nlm.nih.gov/genome/85) (Table 1). An additional set of canine sj-TREC primers were designed upstream (sense) and downstream (antisense), allowing a nested qPCR assay to be performed to increase sensitivity. Finally, primers and probe for a region of the canine albumin gene were designed (Table 1), to enable normalisation of the sj-TREC values for the number of white blood cells in each sample. The probe for the albumin qPCR assay was labelled with a different fluorescent marker (JOE), to that of the sj-TREC probe (6FAM), to enable multiplexing of the two reactions.

**Table 1:** Primers and Taqman probes used in the study.

Method	Primer	Sequence (5’-3’)	Amplicon size (bp)
sj-TREC pre-quantification PCR	Sense	GGCAGCTCTGCATAGTGTGA	364
	Antisense	AGGAAGACCCAGTGCAACAC	
sj-TREC qPCR	Sense	GAGGGAAGGAGGGCAGACCTTCCCCAG	204
	Antisense	GCCAGCTGCAGGGTTTTGG	
	Probe	6FAM-CACGGTGATATGCAGGCAGCTGC-BHQ1	
Albumin qPCR	Sense	CACTTGTTGAACTGCTGAAAC	102
	Antisense	CAGCTGCGCAGCACTTCTC	
	Probe	JOE-CAAGCCCAAGGCAACAGATGAACA-BHQ1	

### Amplification of sj-TREC prior to quantification by real-time qPCR (pre-quantification PCR)

Genomic DNA was extracted from EDTA blood samples using the GenElute Blood Genomic Kit (Sigma-Aldrich, Poole, UK) according to the manufacturer’s instructions. PCR was performed in 20 μl reactions using Immolase DNA polymerase (Bioline), with 2 μl genomic DNA as the template and 1.6 μl of 10 pmol/μl primers. Thermocycling conditions consisted of an initial polymerase activation at 95°C for 10 min, followed by 10 cycles of 94°C for 40 s, 60°C for 30 s and 72°C for 1 min with a final extension step of 72°C for 10 min (G-Storm GS1 thermal cycler, GRI).

### Real-time quantitative PCR (qPCR)

A multiplex real-time qPCR was performed, using the StepOne^™^ Real-Time PCR System (Applied Biosystems 2010 Life Technologies Corporation, Grand Island, USA), to quantify sj-TREC and albumin expression in canine DNA samples that had undergone the pre-quantification PCR.

Primers were initially reconstituted at 200 pmol/μl in molecular biology grade water, while probes were reconstituted at 50 pmol/μL. These were then mixed to a final concentration of 10 pmol/μl for the primers and 2.5 pmol/μl for the probe. The qPCR reaction mixture contained 1 μl of each primer/ probe mix, 10 μl SensiFAST^™^ Probe Hi-ROX Mix (Bioline) and 2 μl template DNA (sj-TREC pre-quantification PCR product), made up to a final volume of 20 μl with molecular biology grade water (Sigma-Aldrich). PCR reactions for each sample were performed in triplicate. The reaction conditions were as follows: 95°C for 5 min, followed by 40 cycles of 95°C for 10 s and 65°C for 60 s. Fluorescent readings were taken after each cycle.

Standard curves were generated from serial dilutions of a recombinant plasmid DNA, containing partial sequences for both canine sj-TREC and albumin to allow quantification of target DNA in the test samples, based on the cycle threshold (Ct) values. Sj-TREC values were normalised for numbers of white blood cells (WBC) (estimated from albumin qPCR values) or numbers of lymphocytes (from haematology data) in the samples, using the following equations:

$${\text{sj-TREC}/1\times 10}^{\text{5}}\text{ WBC }=\;\frac{\text{sj-TREC }\left(\text{copies}/\mu \text{l}\right)}{\text{102}.4}\;\;\times \;\;\frac{{1\times 10}^{\text{5}}}{\left(\text{Albumin }\left(\text{copies}/\mu \text{l}\right)\;\times \;10\right)\;÷\;2}$$

$${\text{sj-TREC}/1\times 10}^{5}\text{ Lymphocytes }=\;\frac{\text{sj-TREC }\left(\text{copies}/\mu \text{l}\right)}{\text{102}.4}\;\;\times \;\;\frac{{1\times 10}^{\text{5}}}{\text{Lymphocytes }\left({\text{10}}^{\text{9}}\text{/L}\right)\;\times \;4000}$$

Where 102.4 is the correction factor applied to adjust for the 10 cycle sj-TREC pre-quantification PCR, the albumin is multiplied by 10 to account for dilution of the gDNA sample into the pre-amplification PCR mix, and the albumin is then divided by 2 because there are 2 copies of the albumin gene per cell. The lymphocyte count is corrected for the amount of blood and the elution volume used in the genomic DNA extraction.

Following initial experiments to validate and optimise the assay, normalisation of sj-TREC against the albumin qPCR values (equivalent to the number of WBC in the sample), was considered more robust, since this could be performed as a single multiplex reaction and would account for any sample degradation that had occurred during storage.

### Statistical analysis

Statistical analyses were performed using a commercial software package (SPSS version 22 for Windows, IBM). Kruskal-Wallis and Mann-Whitney *U* tests were used to compare sj-TREC values between dogs grouped according to age, gender, neuter status and breed type.

## Results

### sj-TREC values decline in Labrador retriever dogs of increasing age

**Fig 1:**
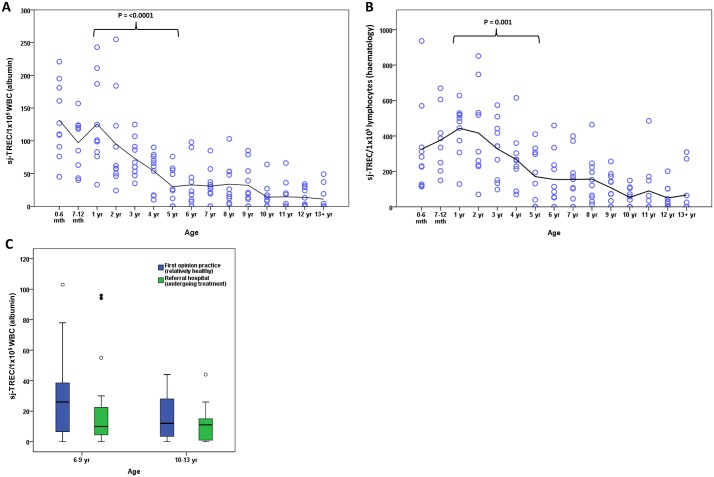
An age associated decline in sj-TREC in Labrador retriever dogs. Sj-TREC values were determined in blood samples (n = 10 per age category; n = 150 total) from Labrador retriever dogs by qPCR and normalised to either (A) white blood cell numbers as calculated by albumin qPCR, or (B) lymphocyte counts from haematological analysis. Each dog is represented by a circle within their age group. A trend line is shown at the mean for each age group. P values were calculated using the Mann Whitney U test. (C) Box and whisker plots are shown to illustrate sj-TREC values grouped according to whether the dogs were undergoing treatment at a veterinary referral hospital or being sampled as part of routine health monitoring in first opinion practices. The horizontal line in each box represents the median, boxes represent the 25^th^ to 75^th^ percentile and whiskers the highest and lowest values which are not outliers. Outliers are represented by open circles and extreme outliers are represented by filled circles.

There was a significant difference comparing dogs of different ages when sj-TREC values were normalised to either WBC numbers, as calculated using the albumin values from qPCR (P<0.0001; Fig 1A), or lymphocyte counts obtained from the haematology results (P<0.0001; Fig 1B). There was marked individual variation in sj-TREC values for dogs of the same age, most noticeable in the younger dogs. A decline in sj-TREC values was observed between the ages of 1 and 5 years (P<0.001). Sj-TREC values then seemed to stabilise between 5–9 years of age, before showing a further gradual decline in the older dogs. In young dogs (<5 years), all the individuals tested had measurable sj-TREC, whereas 16% of mature dogs (5–9 years) and 45% of geriatric dogs (>10 years) demonstrated sj-TREC values below the limit of detection.

The results obtained were generated from a population of dogs undergoing treatment at a tertiary-care veterinary referral hospital. Since these were not healthy dogs, their disease status might be a confounding factor for the age-related effects seen. Therefore, a second group of relatively healthy Labrador retrievers was evaluated that were undergoing routine blood sampling as part of a health monitoring scheme in first opinion practices. Comparison of the sj-TREC values at the age range of 6–9 years and 10–13 years, revealed no significant difference between these two groups of animals (Fig 1C). Additionally, the proportion of dogs with sj-TREC values below the limit of detection was similar in the two groups of dogs. This indicates that the results previously generated using samples from Labrador retrievers undergoing treatment at a veterinary referral hospital appear to be representative of age-related changes in sj-TREC in the general dog population.

### Influence of gender and neutering status on sj-TREC in Labrador retriever dogs

**Fig 2:**
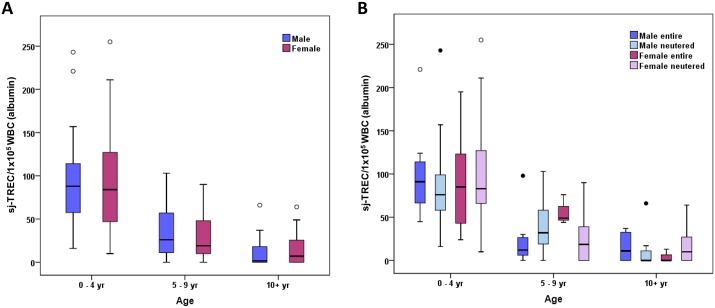
No evidence of gender or neutering status influencing sj-TREC values. Box and whisker plots are shown to illustrate sj-TREC values measured by qPCR in 150 Labrador retrievers of different ages (n = 40–60 per age category) grouped according to (A) gender and (B) reproductive status (neutered vs entire). The horizontal line in each box represents the median, boxes represent the 25^th^ to 75^th^ percentile and whiskers the highest and lowest values which are not outliers. Outliers are represented by open circles and extreme outliers are represented by filled circles.

To examine the effect of gender on the age-associated decline in sj-TREC in Labrador retrievers, male and female dogs were compared. No significant difference in sj-TREC values was observed comparing male and female dogs, when these were grouped into young (0–4 years) mature (5–9 years) or geriatric (>10 years) categories (Fig 2A). The results for each gender were further divided according to neutering status and no significant differences were found (Fig 2B).

### Comparison of the age-related decline of sj-TREC values in large, short-lived breeds and small, long lived breeds of dog

**Fig 3:**
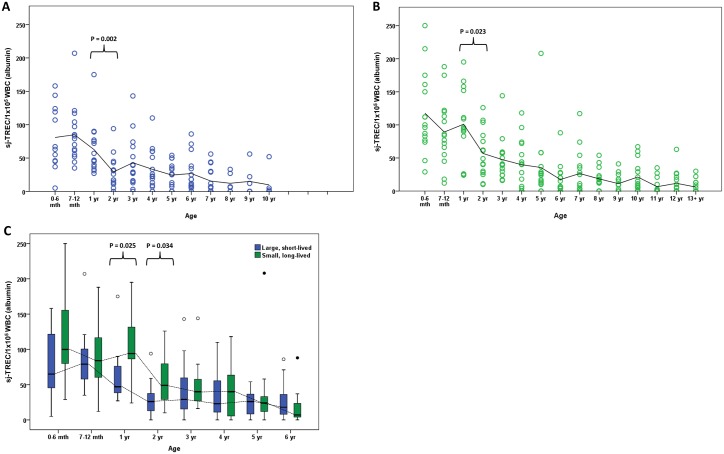
The age associated decline in sj-TREC values differs in short-lived dog breeds compared with long-lived dog breeds. Sj-TREC values were determined by qPCR, using gDNA samples from (A) large, short lived breeds (n = 150) and (B) small, long-lived breeds (n = 225), and normalised to white blood cell numbers, calculated by qPCR for albumin. Each dog is represented by a circle within their age group. A trend line is shown at the mean for each age group. P values were calculated using the Mann Whitney U test. (C) Box and whisker plots are shown to illustrate sj-TREC data from dogs aged 6 years old and under grouped according to age (n = 15 per age group). The horizontal line in each box represents the median, boxes represent the 25^th^ to 75^th^ percentile and whiskers the highest and lowest values which are not outliers. A trend line is shown at the median for each group. Outliers are represented by open circles and extreme outliers are represented by filled circles. P values were calculated using Mann Whitney U tests.

To examine potential breed effects, sj-TRECs were measured in a group of dogs representative of short-lived breeds (Burnese mountain dog, Great Dane and Dogue de Bordeaux) and a group representative of long-lived breeds (Jack Russell terrier and Yorkshire terrier). An age-related decline in sj-TREC values was observed in both groups, similar to that seen in the Labrador retriever dogs (Fig 3A and 3B). The decline in sj-TREC values was most noticeable in dogs under 5 years of age, with significant differences seen between the ages of 1 to 2 years in both short-lived (P = 0.002) and long-lived (P = 0.023) breeds. Significantly lower sj-TREC values were detected in dogs of the short-lived breeds at 1 year (P = 0.025) and 2 years (P = 0.034) of age (Fig 3C), compared with those of the small, long-lived breeds, although there were no differences between breed groups in dogs over 3 years of age. The appearance of dogs with sj-TREC values below the limit of detection was found to occur at a younger age in the short-lived breeds (2 years old) compared with the long-lived breeds (4 years old).

## Discussion

Measurement of sj-TRECs is an established technique for estimating thymic output, which has been employed in a wide range of species, including humans [16], mice [19], primates [28], chickens [17] and pigs [29]. A real-time qPCR assay was developed for measuring sj-TREC in canine blood samples, based on the use of primers and probe adapted from a human assay [27], designed to be specific for the canine orthologue. Normalisation of the sj-TREC values was achieved using either the lymphocyte count or by measuring the reference gene albumin, to provide an estimate of the amount of white blood cell DNA in the sample. Flow cytometry would ideally have been used to obtain T cell counts for the normalisation of sj-TREC values, but fresh samples were not available, and this molecular method has been demonstrated to be a valid alternative approach [27].

When sj-TREC values were assessed in Labrador retriever dogs, normalized against either lymphocyte counts or albumin expression, an age-associated decline was identified in both instances. The greatest decline occurred between the ages of 1 and 5 years, which suggests the largest reduction in thymic output occurs between reaching sexual maturity and early middle age in the canine species. A similar trend is seen in humans, where thymic output remains relatively high until the teenage years, when it begins to decline rapidly [30], with the greatest decline in sj-TRECs having occurred by middle age, between 40 and 50 years old [31]. After reaching 5 years of age, canine sj-TREC values were found to stabilise, with a mean value approximately 20% of that seen in dogs <1 year old, before declining further at around 9 years of age. In older humans, sj-TREC values show a slow decline between the 6^th^ and 9^th^ decades of life before decreasing significantly in the 10^th^ decade [20]. sj-TRECs were undetectable in many mature and geriatric dogs, suggesting that thymic output in these dogs is very low or that production of naïve T-cells has ceased. However, this was not the case in all dogs of a similar age, suggesting that some individuals can maintain thymic output into old age, which is similar to that reported in human studies [20].

Although measurement of sj-TRECs has been widely employed for estimating the age-related decline in RTE, some studies suggest that this technique does not necessarily provide a precise measure of thymic output. Other factors, such as homeostatic proliferation of naïve T cells and their rate of apoptosis in the periphery, can also influence the proportion of sj-TREC positive T cells in blood [18]. There is limited information available on thymic involution in the dog, making it difficult to determine precisely how the sj-TREC data from this study relates to changes in the canine thymus with increasing age. Thymic weight has been shown to increase in dogs between 1 and 6 months of age [32], after which time a reduction in thymic weight seems to correlate with atrophy of the thymic cortex in dogs up to 2 years of age [33], but longitudinal studies of dogs beyond this age are lacking. However, similarities between the profile of sj-TREC in Labrador retriever dogs and that seen in other species suggests that the canine thymus is likely to demonstrate the same the same multi-phasic involution observed in other vertebrates [34]. Thus adding further to the evidence that the age-related decline of the thymus is an evolutionary conserved event [4].

Considerable individual variation in sj-TREC values was observed, comparing dogs of the same age, which was particularly evident in the young dogs. The same finding was reported in a recent study of a small cohort of mixed breed dogs in Japan [21], and is also apparent in some human studies [31, 35]. The individual variation in sj-TREC values within the Labrador retriever breed might offer opportunities for studying genetic factors that are associated with thymic output. It remains to be established whether juvenile dogs that demonstrate relatively low sj-TREC values progress to becoming sj-TREC deficient earlier in life and whether this impacts on their lifespan/healthspan. A premature decline in sj-TREC values has been associated with susceptibility to human diseases such as amyotrophic lateral sclerosis [36], juvenile idiopathic arthritis [37] and renal disease [38]. However, without performing longitudinal studies it is not possible to determine how sj-TRECs change with time in individual dogs, and the influence this might have on susceptibility to disease and lifespan.

Most of the samples used for the study were from dogs receiving treatment at a tertiary-care veterinary referral hospital, and although cases were carefully selected that were ‘haematologically healthy’, it is possible that the disease status of the animal might have impacted on sj-TREC values. To investigate this further, a second population of older Labrador retrievers, that were undergoing routine blood sampling and testing as part of a ‘senior wellness program’ in first opinion practices, were recruited to the study. There was no significant difference in the sj-TREC values comparing the dogs undergoing treatment at a referral hospital, and those dogs attending first opinion practices, suggesting that the hospital cases used for the sj-TREC analysis were representative of the wider Labrador retriever population.

Several studies in humans have demonstrated gender-related differences in the decline in thymic output with age [20, 35], whereby females have been found to have relatively higher sj-TREC values than males. Similarly, male mice appear to undergo thymic atrophy more rapidly than female mice [39]. Our study did not identify any gender-related differences in canine sj-TREC values, but this might be explained by the inclusion of animals which have undergone surgical neutering [40]. However, no significant difference in sj-TREC values was observed between neutered and sexually intact dogs of either gender, although the relatively low number of non-neutered dogs in the older age groups might have influenced this result. Castration of male mice and humans has been shown to cause an improvement in thymic function [41], while elevated levels of oestrogen are known to enhance thymic atrophy in females [42]. Studies in dogs have shown that reproductive status can affect lifespan, with those left intact having a shorter lifespan, compared with those that have been neutered [43]. However, this variable is likely to play a complex role and neutering has also been shown to increase the incidence of some diseases in later life [44].

Breed influences on sj-TREC were investigated by studying two groups that represent the extremes of the canine lifespan spectrum; small breed dogs with a relatively long life expectancy and large breed dogs with a relatively short life expectancy. Studies in humans have proposed an association between maintenance of thymic function and lifespan [45] and have suggested that sj-TREC analysis might be of use as a biomarker for determining longevity [20]. Both groups demonstrated a similar age-related decline in sj-TREC, with the greatest reduction occurring between young adulthood and middle age. However, the onset of the decline in sj-TRECs was found to occur at an earlier age in the short-lived breeds compared with the long-lived breeds, suggesting that thymic involution might occur prematurely in the former. Furthermore, some individuals of short-lived breeds were identified that had undetectable sj-TREC as young as 2 years of age, compared with the other breeds assessed, where this did not occur until around 4–5 years of age. Therefore, if thymic involution is occurring at an earlier biological time point in some dog breeds, this might have an impact on their subsequent lifespan/healthspan. This is consistent with a recent study [25] in which there was a strong relationship between lifespan, body size and rate of aging, with the largest breeds also having evidence of an earlier onset of the aging process.

Breed differences in biological aging and immunosenescence are likely due to genetic factors, some of which might impact on thymic health and T cell output, either intrinsically or extrinsically. Genes that influence the structure of the thymus or impact on the thymic microenvironment could potentially be involved [46]. Interleukin 7 (IL-7) has been shown to have a major influence on T cell development in the thymus and in controlling thymic output [47]. Additionally, the production of IL-7 in the mouse thymus has been shown to decline with age [48], suggesting it might play a role in thymic involution. One possible extrinsic mechanism might involve the growth hormone (GH)/ insulin-like growth factor 1 (IGF-1) axis. This has been shown to play a role in regulating aging and life-span in several species [49, 50], and is also implicated as the major genetic factor contributing to differences in size between dog breeds. [51–53]. Since both GH and IGF-1 have been shown to play a role in thymic function [54], it would be interesting to investigate whether breed differences in these hormones might also influence thymic size, output and involution, associated with the rate of decline in sj-TREC observed.

In summary, we have demonstrated an age-associated decline in sj-TREC in several dog breeds. The presence of relatively young dogs that are sj-TREC deficient, suggests that premature thymic involution might be occurring in short-lived breeds. The intra-breed and inter-breed differences in thymic output offer the opportunity to use the dog as a model to study genetic factors that influence thymic health. Furthermore, the relationship between thymic output and the response to vaccination or susceptibility to disease remains to be established.
